# Is there a healthy-food premium? A cross-sectional analysis of the UK online food delivery sector

**DOI:** 10.1093/heapro/daag126

**Published:** 2026-08-17

**Authors:** Yuma Asai, Laura Cornelsen, Alexandra Kalbus

**Affiliations:** Department of Public Health, Environments and Society, London School of Hygiene & Tropical Medicine, 15-17 Tavistock Place, London WC1H 9SH, United Kingdom; Department of Public Health and Health Policy, Graduate School of Medicine, The University of Tokyo, Hongo 7-3-1 Bunkyo-ku, Tokyo 113-0033, Japan; Department of Public Health, Environments and Society, London School of Hygiene & Tropical Medicine, 15-17 Tavistock Place, London WC1H 9SH, United Kingdom; Department of Public Health, Environments and Society, London School of Hygiene & Tropical Medicine, 15-17 Tavistock Place, London WC1H 9SH, United Kingdom

**Keywords:** online food delivery, digital food environment, food prices, kilocalories, health inequalities, out-of-home food

## Abstract

Online food delivery (OFD) platforms account for a growing share of out-of-home meals, but their often poor nutritional quality may worsen diets and related health inequalities. Furthermore, pricing structures within this sector may be systematically incentivizing the selection of higher-calorie items. Using menu data collected in June 2023 from two major UK OFD platforms, this study examined 7392 unique adult menu items from large restaurant chains. The price–calorie relationship (per 100 kcal) was modelled using restaurant-chain fixed-effects regressions controlling for menu categories. Results indicated a significant nonlinear relationship: while price initially increased with calorie content (101.14 pence per additional 100 kcal, 95% CI [87.98, 114.29]), the rate of increase diminished by 2.19 pence per (100 kcal)^2^ (95% CI [1.50, 2.87]), suggesting a relative discount for higher-calorie options. Furthermore, menu items classified as ‘healthier’ (<600 kcal, consistent with recommendation on calorie content per meal in England) were priced relatively higher, costing 70.3 pence more per 100 kcal than items containing ≥600 kcal (95% CI [61.9, 78.8]) after controlling for menu categories. These findings suggest that pricing structures within the UK OFD sector financially favour higher-calorie options. Policy makers should consider strategies addressing pricing mechanisms to ensure healthier options are equally accessible.

Contribution to Health Promotion

- We investigated the relationship between calorie content and price of food items from UK chain restaurants on online food delivery platforms.
- There was a nonlinear relationship between calorie content and price, with the rate of price increases slowing as calorie content rises.
- ‘Healthier’ menu items (<600 kcal) cost 70.3 pence/100 kcal more than items ≥600 kcal.
- This suggests that pricing structures provide an economic incentive for higher-calorie, potentially unhealthy foods.

## Introduction

A suboptimal diet is defined in the Global Burden of Disease Study by dietary risk factors, such as low intake of whole grains, fruits, and vegetables, together with high intake of sodium, and is a leading risk factor for the global disease burden, accounting for an estimated 11 million deaths annually (GBD 2017 Diet Collaborators 2019). This risk is disproportionately concentrated among socioeconomically disadvantaged groups, exacerbating health disparities (Darmon and Drewnowski 2008, Giskes *et al*. 2010). In the UK, the prevalence of obesity and diet-related diseases follows a socioeconomic gradient (Maguire and Monsivais 2015, Bann *et al*. 2017). This gradient mirrors structural inequalities in the physical food environment, where fast-food outlet density is significantly higher in deprived areas—a disparity that widened by 28% between 2011 and 2024 (Boskovic *et al*. 2026). While food environment research has traditionally focused on physical settings, the rapid growth of online platforms has given rise to a digital food environment closely interconnected with its physical counterpart (Granheim *et al*. 2022). This digital environment follows a broadly similar socioeconomic gradient: the number of accessible online food outlets is 50% greater in the most deprived neighbourhoods than in the least deprived (Keeble *et al*. 2021), and the deprivation–exposure association varies geographically (Kalbus *et al*. 2023).

The online food delivery (OFD) sector, defined here as aggregator platforms facilitating the purchase and delivery of prepared food (Fernandez and Raine 2021, Jia *et al*. 2024), excluding online groceries, direct telephone orders, and orders placed through restaurants’ own websites, has grown, particularly since the COVID-19 pandemic. These platforms aggregate menus from a wide range of food outlets, including national restaurant chains, independent takeaways, and dessert and coffee shops, thus providing access to diverse cuisine types (Keeble *et al*. 2021). In the UK, the online food ordering and delivery platform market was estimated at £3.8 billion in 2024 (IBISWorld 2025), and the proportion of adults reporting OFD use at least once in the past 7 days increased from 19% in 2018 to 28% in 2021 (Gupta *et al*. 2024). However, foods available on OFD platforms are frequently calorie dense and nutrient poor (Jia *et al*. 2024). The average energy content of takeaway meals varies by cuisine, from ∼1125 to 1820 kcal (Jaworowska *et al*. 2014), and an analysis of UK chain restaurants indicated that 47% of main meals exceed 1000 kcal (Robinson *et al*. 2018), far above the government-recommended 600 kcal per lunch or dinner (Public Health England 2018).

Despite these nutritional issues, price remains a primary determinant of food choices (Lennernäs *et al*. 1997, Glanz *et al*. 1998, Fernqvist *et al*. 2024). Furthermore, marketing tactics, such as frequent discounts, may incentivize the selection of calorie-dense items (Jia *et al*. 2024). In the UK, more than 65% of restaurants on a major delivery app offer price promotions, with even higher rates in socioeconomically disadvantaged areas (Huang *et al*. 2025). In retail settings, calorie-dense foods have been reported to be cheaper per unit of energy than nutrient-dense foods (Drewnowski and Specter 2004, Jones *et al*. 2014). Analysis of the consumer price index data in the UK showed that in 2023 healthier foods cost £0.81 per 100 kcal, compared with £0.33 per 100 kcal for less healthy foods (Hoenink *et al*. 2024). This premium creates an economic barrier to accessing a healthier diet, particularly for low-income populations (Drewnowski and Darmon 2005, Darmon and Drewnowski 2015). However, the extent to which these price-nutrient trade-offs observed in retail markets also exist in the growing OFD market in the UK has not been investigated. Understanding OFD pricing structures is critical as user demographics suggest that the sector may be contributing to widening health inequalities. In England, households in lower social grades are more than twice as likely to be using takeaway food delivery apps compared with households in the highest grade (Cummins *et al*. 2024). Consequently, if healthier options on these platforms carry a price premium, the pricing structure could disproportionately affect lower socioeconomic groups, widening diet-related health inequalities.

Although England implemented restrictions on volume-based price promotions (e.g. multibuy offers) for products high in fat, salt, and sugar in retail settings in October 2025, the out-of-home (OOH) food sector is exempt from these restrictions, including when OOH food is purchased via online apps or aggregator platforms (Department of Health and Social Care 2023). As a result, OFD platforms are exempt from comparable controls on price promotions. This regulatory asymmetry suggests that extending similar restrictions to the digital OOH sector could be considered; however, the lack of empirical evidence may hinder the development of such interventions. Such evidence can, however, only be generated for large businesses, typically chains: mandatory kilocalorie labelling in England, implemented in April 2022, applies only to businesses with 250 or more employees (Department of Health and Social Care 2021). Hence, item-level calorie information is systematically available and price–calorie relationships can be characterized at scale only for large chains. This study therefore (i) quantified the relationship between price (per item and per 100 kcal) and calorie content across large restaurant chains, accounting for chain-specific heterogeneity, and (ii) assessed whether a price premium exists for menu items defined as those containing <600 kcal per item, in line with recommendation on healthy eating in England (Public Health England 2018).

## Materials and methods

### Data acquisition and processing

This study used secondary data without human participants and was approved by the Ethics Committee of the London School of Hygiene & Tropical Medicine, University of London (approval number 32228/RR/37597).

**Figure 1:**
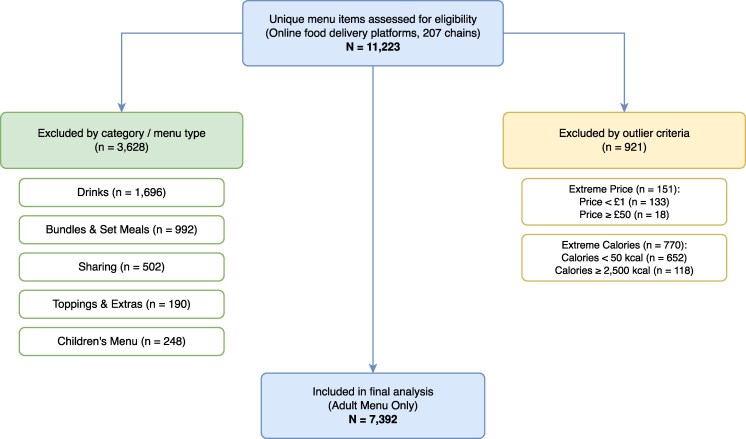
Data cleaning and sample construction. Flow diagram showing the inclusion and exclusion of unique menu items assessed for eligibility and the derivation of the final analytical sample (*N* = 7392) from the initial set (*N* = 11 223). Note: the sum of exclusions by category (*n* = 3628) and outliers (*n* = 921) exceeds the total number of removed items (*n* = 3831) because 718 items met multiple criteria; kcal = kilocalories; £ = pounds sterling.

Data acquisition and cleaning are summarized in Fig. 1. Online menu data were obtained from the websites of Deliveroo and Uber Eats in June 2023 using automated collection tools (Greener 2022, 2023) that captured restaurant information (name, tag, location) and item details (name, description, price, item category, calorie content in kilocalories). Data were collected by querying each platform for every small area in Great Britain: each Lower layer Super Output Area in England (*n* = 32 844) and Wales (*n* = 1909) and each Data Zone in Scotland (*n* = 6976), giving complete coverage (41 729 small areas, of which 88.7% had OFD available). The price variable is the single listed price shown for each item at the time of data collection. This displayed price may be either a standard price or a temporarily reduced or promotional price; the collection tools recorded only one price per item and could not distinguish between them, nor capture order- or basket-level promotions, personalized offers, or platform-level charges (e.g. delivery and service fees). The data-collection procedure followed our earlier protocol (Kalbus *et al*. 2023), and both collection tools are publicly available and cited (Greener 2022, 2023); the full keyword dictionary used for classification is provided in Supplementary Appendix A.

This study focused on large restaurant chains, i.e. businesses with 250 or more employees, which have been subject to mandatory calorie labelling under the Calorie Labelling (Out of Home Sector) (England) Regulations 2021, implemented in April 2022 (Department of Health and Social Care 2021). Our analysis focuses on large chains, in line with previous research (Robinson *et al*. 2018, Muc *et al*. 2019), because standardized energy information was systematically available only for these chains. They are further policy relevant as the target of the UK Government’s subsequently announced ‘healthy food standard’ (Discussion). Restaurant chains were defined as outlets listed on the MenuTracker database (Huang *et al*. 2022) or those appearing at least five times in the dataset and offering 10 or more items with calorie information. Because menu data were captured for many small areas across Great Britain, popular chain items were recorded repeatedly across the numerous branches of the same chain. To prevent items from being over-represented, a ‘representative menu’ likely to be encountered by consumers around Great Britain was therefore constructed for each chain by retaining only items offered by at least two branches and consolidating these location-level records into a single chain-level menu. Items appearing at a single branch only, e.g. local specials, were excluded. This yielded a dataset of 11 223 unique items for eligibility assessment.

### Classification of item categories

Items were classified into 10 categories using a term-level keyword dictionary (the full keyword list is provided in Supplementary Appendix A). The categories were defined bottom-up from the platforms’ item category and tag fields (Data acquisition and processing) and a name-and-description keyword dictionary informed by industry sector reports (Deliveroo 2024, Uber Eats 2024), rather than from a predefined scheme. The resulting groupings are consistent with prior analyses of UK chain-restaurant menus (Robinson *et al*. 2018, Muc *et al*. 2019). Classification was rule based, deterministic, and fully reproducible, not an automated classifier or AI model. Instead, generative AI (Google Gemini) was used only to suggest candidate synonyms, all reviewed and approved by the lead author. It proceeded in two steps: items were first assigned from the derived-type indicators in the source data (e.g. pizza, burger, dessert, drink, sharing, and bundle), and any not captured were then matched on their name and description against the dictionary, with the residual retained as ‘other’. These 10 categories were defined as main dishes, desserts, drinks, bundles and set meals, sharing, salads and bowls, soups, sides and snacks, toppings and extras, and other. Supplementary Appendix A documents 23 dish-level subcategories, including separate burger, pizza, fried chicken, rice dish, and noodle and pasta groupings, together with their full mapping to the 10 overarching categories above. For the main analysis, these subcategories were collapsed into the 10 categories so that the menu-category fixed effects remain interpretable and are estimated based on sufficient subgroup sample sizes.

### Exclusion criteria and final sample construction

Items were excluded at the category level if deemed unsuitable for the analysis of individual food items (*n* = 3628). Excluded categories were bundles and set meals (not individual items), sharing items (likely to be consumed by more than one person), toppings and extras (not consumed on their own), and drinks. Drinks were excluded because their price–calorie relationship is structurally different compared to solid foods (e.g. zero-calorie and sugar-sweetened beverages often share similar price points or may be externally influenced by the Soft Drinks Industry Levy that applies to sugar-sweetened beverages with over 5 g of sugar per 100 g). Children’s menu items (*n* = 248) were also excluded. Further 921 items were excluded as outliers following established practice in the literature, based on price and calorie thresholds: price < £1 (*n* = 133) and ≥£50 (*n* = 18) (Huang *et al*. 2025) or extreme calorie content < 50 kcal (n = 652) and ≥2500 kcal (*n* = 118) (Olea López and Johnson 2016, Huang 2018). The great majority of items above this threshold were sharing items or multiperson set-meal bundles, and the remainder (*n* = 21) were very large single products, predominantly family-sized pizzas (examples are provided in Supplementary Appendix I). Figure 1 summarizes the final analytical sample (*N* = 7392 across 200 restaurant chains) after accounting for overlapping exclusion criteria (*n* = 718).

### Analytical strategy

#### Outcome and exposure measures

The primary outcome was menu item price, analysed as (i) absolute price per item (pence) and (ii) price per 100 kcal (pence). The primary exposure was calorie content, scaled to 100 kcal units. To assess price premium for healthier options, a binary indicator was created, classifying items with <600 kcal per item as ‘healthier’, or less healthy if exceeding 600 kcal following recommendation on a single meal’s calorie content in England (Public Health England 2018).

#### Statistical analysis

Data management and statistical analyses were performed using Stata/MP 18.0. Due to right-skewed distributions, descriptive statistics are reported as medians with interquartile ranges (IQRs). Restaurant-chain fixed-effects (FE) models were used to control for unobserved heterogeneity, with standard errors clustered at the chain level (see Supplementary Appendix B for the price–calorie model specifications). The primary regression estimated the price–calorie relationship. Because the observed relationship between price and calories was nonlinear, a quadratic calorie term was included. To investigate heterogeneity by food type, interactions between calorie content and the six retained categories were added. From the interaction model, predicted prices were calculated at representative category calorie levels (25th, 50th, 75th percentiles) to visualize heterogeneity. Finally, category-specific ‘turning points’ were calculated using the standard vertex formula $-\beta_{1}/(2\beta_{2})$ based on the estimated coefficients from the FE models. To estimate price differences between higher- and lower-calorie content menu items, chain FE models regressed price per 100 kcal on the binary ‘healthier’ indicator (<600 kcal) while controlling for menu categories (see Supplementary Appendix C for model specifications). Interactions with item categories were used to test for effect heterogeneity. Statistical significance was defined as *P* < .05.

#### Sensitivity analysis

To test robustness to the chosen outlier thresholds, primary models were re-estimated using an expanded sample that included items with up to 3000 kcal (*n* = 18). This threshold was selected based on data exploration indicating that items exceeding 3000 kcal were predominantly labelled as sharing items.

## Results

### Sample characteristics

Table 1 presents descriptive statistics on price, calorie content, and item category composition for the final analytical sample (*N* = 7392). Overall, 40.7% of items, including half of main dishes (50.8%), contained ≥600 kcal. Price disparities across categories were also evident; salads and bowls and soups exhibited the highest median prices per 100 kcal (£2.00 and £2.47, respectively), whereas sides and snacks and desserts had the lowest median price per 100 kcal (£1.01 and £1.06, respectively).

**Table 1:** Descriptive statistics for menu items (*N* = 7392).

Category	*N*	Price (£)	Calories (kcal)	Price per 100 kcal (£)
		Median [IQR]	Median [IQR]	Median [IQR]
Overall	7392	7.12 [4.50, 10.80]	518 [336, 784]	1.33 [0.95, 1.90]
Calorie classification				
<600 kcal	4380	5.30 [3.50, 7.75]	368 [253, 472]	1.54 [1.05, 2.29]
≥600 kcal	3012	10.49 [7.50, 13.62]	846 [710, 1071]	1.15 [0.86, 1.48]
Item category				
Main dishes	3745	8.95 [6.00, 12.95]	613 [417, 891]	1.43 [1.07, 1.94]
Desserts	1494	4.80 [3.00, 7.40]	430 [301, 688]	1.06 [0.75, 1.42]
Sides and snacks	427	4.99 [3.75, 7.00]	472 [324, 685]	1.01 [0.77, 1.50]
Salads and bowls	345	8.00 [5.25, 11.50]	412 [257, 585]	2.00 [1.38, 2.92]
Soups	144	9.50 [5.40, 12.50]	355 [216, 558]	2.47 [1.76, 3.34]
Other^a^	1237	5.25 [3.55, 7.79]	403 [245, 605]	1.33 [0.90, 1.99]

Note: IQR = interquartile range (25th–75th percentiles).

^a^The ‘other’ category is a heterogeneous residual group of items that the deterministic rules did not assign to any single standard category; it includes composite or cross-cuisine dishes, as well as stand-alone condiments, sauces, and accompaniments, and is a residual grouping rather than a claim about any single food type (see Supplementary Appendix H).

### Price–calorie relationship

The FE model, including restaurant-chain and menu-category fixed effects, revealed a significant nonlinear relationship between price and calories (Table 2). The linear term was positive (β = 101.14 pence per 100 kcal; 95% CI [87.98, 114.29]), while the squared term was negative (β_2_ = −2.19; 95% CI [−2.87, −1.50]), consistent with a concave (inverse U-shaped) relationship.

**Table 2:** FE regression estimates for the relationship between price (in pence) and calories.

Predictor	Coefficient (pence)	95% confidence interval	*P* value
Calories (per 100 kcal)	101.14	[87.98, 114.29]	<.001
Calories (per 100 kcal) squared	−2.19	[−2.87, −1.50]	<.001
Item category			
(Ref: desserts)			
Main dishes	266.43	[222.85, 310.01]	<.001
Sides and snacks	2.16	[−42.04, 46.37]	.923
Salads and bowls	276.79	[227.26, 326.31]	<.001
Soups	342.05	[255.45, 428.65]	<.001
Other	80.83	[45.06, 116.60]	<.001
Constant	139.47	[81.42, 197.51]	<.001

Notes: dependent variable: price in pence. Calories scaled to 100 kcal. Models include chain FE with standard errors clustered by chain.

**Figure 2:**
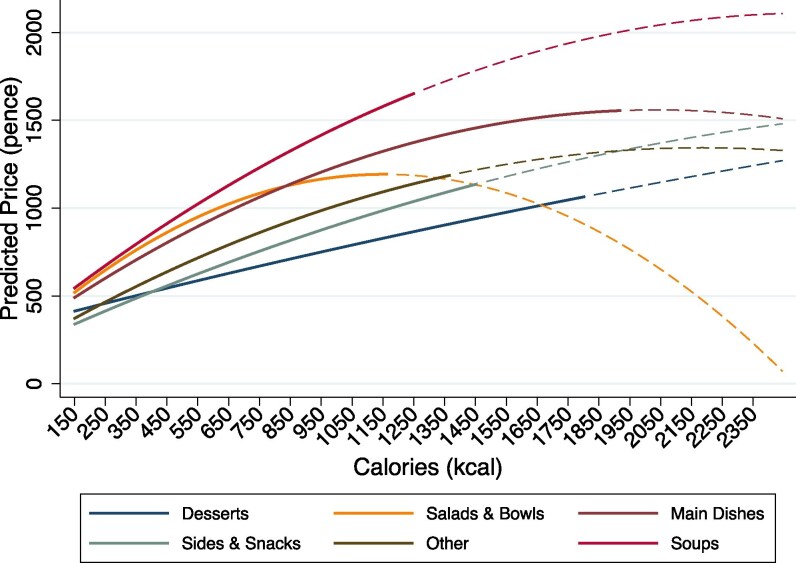
Predicted price as a function of calories, stratified by item category. Category-specific curves show model-based predicted prices (pence) across calories (kcal) from a nonlinear FE specification with a category interaction, with standard errors clustered by chain. Note. Solid lines indicate predictions within the observed calorie range for each category (up to the category-specific 99th percentile); dashed lines show model extrapolations beyond the observed range; kcal = kilocalories.

The shape of this nonlinear relationship differed significantly across item categories [Fig. 2; cluster-robust *F*-test: *F*(10, 199) = 9.24, *P* < .001; see Supplementary Appendix E for the full interaction model estimates]. For all menu items overall, the estimated turning point up to which the price increases (at a decreasing rate) as items become more calorific was 2309 kcal. In category-specific estimates from the interaction model, the turning point occurred at 1151 kcal for salads and bowls and 2033 kcal for main dishes, which accounted for 52% of the analytical sample. For soups, desserts, sides and snacks, and other, the squared interaction terms were not statistically significant (*P* > .05), and category-specific concavity was not detected within the observed calorie range.

**Figure 3:**
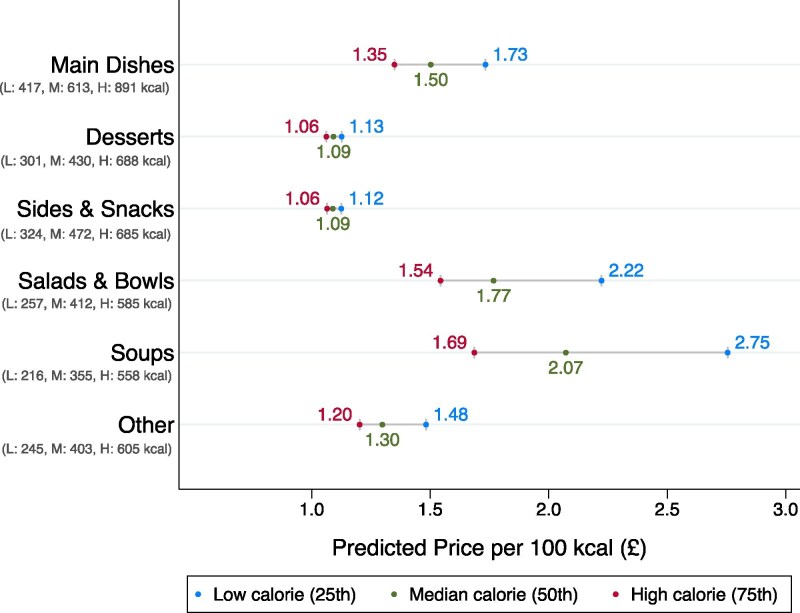
Predicted price per 100 kcal (£) for each item category evaluated at the category-specific low (25th), median (50th), and high (75th) percentiles of calories (kcal). Note: abbreviations in parentheses on the y-axis denote calorie percentiles: L = low (25th percentile), M = median (50th percentile), H = high (75th percentile), kcal = kilocalories.

Figure 3 displays predicted price per 100 kcal at category-specific low (25th percentile), median (50th percentile), and high (75th percentile) calorie levels, demonstrating a stronger gradient of decreasing cost per calorie particularly for main dishes, salads and bowls, and soups.

### Price differences by healthiness indicator

Descriptive analysis revealed that while items classified as ‘healthier’ (<600 kcal) had a lower median absolute price (£5.30) compared to items ≥600 kcal (£10.49), they exhibited a higher median price per 100 kcal (£1.54 vs. £1.15) (Table 1).

In the main-effects chain FE model (without interactions), items containing <600 kcal were priced 70.3 pence higher per 100 kcal than items with ≥600 kcal (95% CI [61.9, 78.8]; *P* < .001; see Supplementary Appendix D for the full main-effects model estimates), controlling for menu categories.

**Figure 4:**
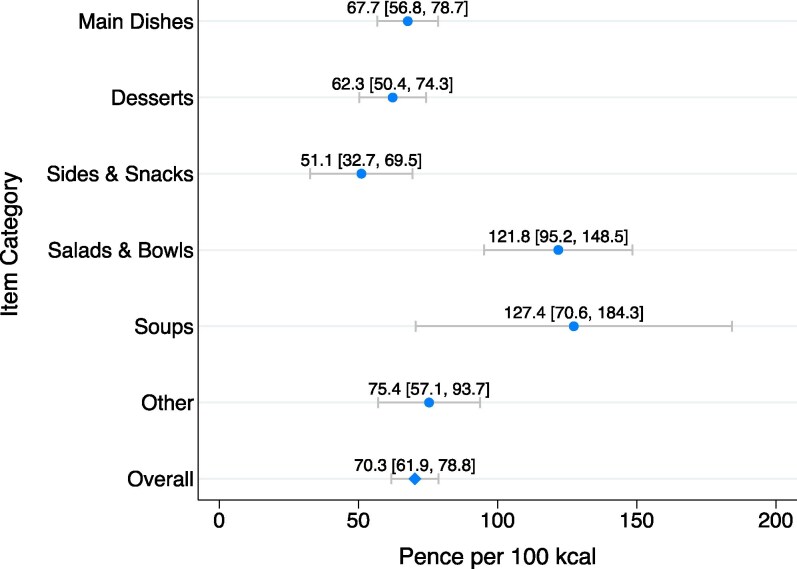
Estimated mean price difference (pence per 100 kcal) between <600 kcal items and ≥600 kcal items for each category and the overall sample, with horizontal bars indicating 95% confidence intervals. Note: kcal = kilocalories.

Interaction analysis indicated that the estimated price difference between items <600 kcal and ≥600 kcal varied by item category [*F*(5, 199) = 6.35, *P* < .001]. As shown in Fig. 4 (see Supplementary Appendix F for category-specific interaction coefficients), the mean price difference was most pronounced for soups and salads and bowls, which exhibited a significantly larger price difference between healthier and less healthy items averaging around £1.22–£1.27 per 100 kcal. Conversely, price differences in main dishes, sides and snacks, and other category were statistically indistinguishable from the difference in desserts (base) averaging between 51 and 75 pence per 100 kcal.

### Sensitivity analysis

When the exclusion threshold was extended from 2500 to 3000 kcal (*n* = 18 items added), the inverse U-shaped relationship persisted (linear coefficient: 96.74 pence per 100 kcal; quadratic coefficient: −1.88 pence per (100 kcal)^2^, *P* < .001), and the estimated turning point shifted to 2573 kcal. In a further sensitivity analysis, the models were re-estimated on a combined sample that re-included the previously excluded bundles, set meals, and sharing items (1275 items added; *n* = 8667 items in total). The findings were materially unchanged: the inverse U-shaped price–calorie relationship persisted, and items below 600 kcal remained more expensive per 100 kcal than those at or above 600 kcal (74.13 pence; 95% CI [64.7, 83.5]). Full results for the combined sample, and for bundles, set meals and sharing items analysed separately, are reported in Supplementary Appendix G of the Supplementary File.

## Discussion

### Summary of findings

This study, using large-scale menu data from major UK OFD platforms, observed a decreasing rate of price increase as calorie content rose, implying that consumers choosing lower-calorie options face highest cost per calorie. Overall, 41% of items analysed exceeded 600 kcal recommended per meal (Public Health England 2018), including 52% of main dishes. Menu items below the energy content of 600 kcal were, on average, 70.3 pence (95% CI [61.9, 78.8]) more expensive per 100 kcal compared to items containing ≥600 kcal, including 67.7 pence (95% CI [56.8–78.7]) for main dishes.

### Interpretation of findings

The observed pricing structure suggests a misaligned incentive, consistent with the caloric excess previously documented in the UK restaurant sector (Robinson *et al*. 2018, Muc *et al*. 2019). Of the 27 chain restaurants analysed by Robinson *et al*. (2018), 91% of main meals exceeded 600 kcal, compared with 52% of main dishes in the present study. Whether this represents an improvement over time is uncertain, as the two studies differ in their unit of analysis. Robinson *et al*. (2018) sampled complete meals (e.g. a burger served with chips) and explicitly excluded individually sold items, whereas the present study analysed individual menu items. While the nonlinear relationship between price and calories varied by category, it was prominent among main dishes, which comprise approximately half of the menu offerings. Across the calorie distribution, the predicted price per 100 kcal decreased consistently (Fig. 3), indicating that higher-calorie items offer progressively greater value. This pattern is consistent with the estimated quadratic relationship, whose turning points were 2309 kcal overall and 2033 kcal for main dishes; the higher overall estimate reflects the inclusion of more linear categories, such as sides and snacks and desserts (see Supplementary Appendix E). In contrast, the price–calorie trajectory for salads and bowls peaks earlier (turning point: 1151 kcal), reflecting the narrower calorie range and lower intrinsic energy density of these items. Concurrently, the ‘health premium’, whereby items below the 600 kcal threshold are more expensive per calorie compared to higher-calorie items, was most pronounced for soup £1.27 (95% CI [0.71–1.84]) and salads and bowls £1.21 (95% CI [0.95–1.49]).

The inverse relationship between calorie density and price per calorie is a documented structural feature of global retail food markets (Drewnowski and Specter 2004, Drewnowski and Darmon 2005, Darmon and Drewnowski 2015). The relatively higher cost of fruits and vegetables has been suggested as one driver of the higher cost of healthier diets (Hoenink *et al*. 2024, Herforth *et al*. 2025). This economic dynamic extends to the traditional food service sector, where nonproportional or ‘value-size’ pricing can incentivize larger, calorie-dense portions (Vermeer *et al*. 2010). Emerging international evidence suggests that these misaligned incentives are also present in the digital food environment. Research from India (Abdulkader *et al*. 2022), New Zealand (Mahawar *et al*. 2022), Australia (Wang *et al*. 2021), and Brazil (Horta *et al*. 2021) documented lower nutritional quality in less expensive OFD menus and promotion-heavy marketing for unhealthy items. The present analysis indicates that the per-calorie price discount for higher-calorie items is also a structural feature of the UK’s digital food sector.

A comparable price penalty for healthier options has been reported in UK supermarkets, where less healthy foods cost less per 100 kcal than healthier foods (33 vs. 81 pence; a 48-pence gap) (Hoenink *et al*. 2024). The figures are not, however, directly comparable. Supermarkets predominantly offer items intended to be used to prepare meals, alongside prepared food, while our estimates cover only ready-to-eat prepared meals from the OOH food sector. The two comparisons also define ‘healthier’ differently—the supermarket figures contrast foods grouped by nutrient profiling, whereas ours contrasts items below versus at or above 600 kcal. The two settings are therefore best interpreted as sharing a direction of effect rather than a direct comparison.

To some extent, decreasing cost per additional calorie is expected as nonfood costs associated with a menu item, such as labour and rent, may not scale proportionally with calorie content. This may partly explain the observed volume discount, whereby higher-calorie items are relatively less expensive than lower-calorie dishes. In addition, fresh ingredients with low-calorie density may incur higher base costs, such as storage and preparation, thereby increasing their relative cost compared to items with a higher energy density and/or comprising ultraprocessed ingredients, which lower the unit cost per calorie. For instance, ultraprocessed foods (UPFs) tend to be cheaper than minimally processed foods (MPFs) on a per-calorie basis, with research from Belgium reporting mean prices of €0.55 per 100 kcal for UPFs compared to €1.29 for MPFs (Vandevijvere *et al*. 2020).

Taken together, the availability of higher-calorie OOH meals is coupled with a pricing architecture that financially disincentivizes lower-calorie selections and may constitute an economic barrier to healthier diets. Such pricing structures may widen diet-related health inequalities: OFD use is more common among households with lower socioeconomic status (Cummins *et al*. 2024), and consumers under economic constraint tend to choose lower-quality diets because they cost less per calorie (Drewnowski and Darmon 2005, Darmon and Drewnowski 2015). Pricing may therefore be particularly important for lower-income consumers, as a premium on lower-calorie items takes a larger share of a smaller food budget. Nor is income the only dimension along which this burden is likely to vary: app use is concentrated among younger adults (Cummins *et al*. 2024) and households with children (Keeble *et al*. 2020), and UK users report limited time to cook, including from childcare and work, as a key reason for use (Keeble *et al*. 2022). People with limited mobility may also rely on delivery as an important means of food access (Hwang *et al*. 2024) and so have little scope to substitute towards cheaper alternatives, meaning a premium on healthier options may weigh most heavily on them. Because the menu-level data carry no information on who buys these items, these distributional effects are presented as plausible equity concerns.

### Limitations

This study has several limitations. First, data were restricted to major chain restaurants on two leading UK OFD platforms due to calorie labelling availability. While this captures a significant market share, findings may not be generalizable to independent restaurants or other delivery services. Additionally, the presence of ‘virtual brands’ associated with established high-street restaurants may result in an overestimation of distinct chains. Second, the analysis focused on individual menu items rather than complete meal orders. By excluding ‘bundles’ and ‘drinks’, the total calories purchased per transaction may be underestimated, as consumers frequently purchase combinations (e.g. a burger with fries and a soft drink). Therefore, the economic incentives associated with multiproduct meal combinations and bundle discounts may differ from the price structures observed in single-item analyses. To probe this, sensitivity analyses re-including bundles, set meals, and sharing items (reported in the Results section and Supplementary File) were directionally consistent with the main results, although a meaningful per-portion price and calorie value cannot be defined for these multiportion items. Third, the absence of weight or volume data precluded the calculation of price per unit mass. Accordingly, it remains undetermined whether the observed price premiums for lower-calorie items reflect differences in underlying production costs or simply smaller portion sizes. Regardless, pricing structures should not incentivize either greater portion size or greater calorie content per item. Fourth, nutritional assessment was limited to calorie content, without accounting for ingredients or macronutrient and micronutrient composition. Without these indicators, any comparison between healthier and less healthy items remains partial. Fifth, keyword-based classification may introduce misclassification bias, particularly in the ‘salads and bowls’ category, where it was not always possible to distinguish between items intended as mains or sides. Sixth, as detailed in the Methods section, only a single listed price per item was available; standard prices could not be separated from temporary or promotional prices, and order-level promotions, personalized offers, and platform-level costs were not captured. Where the captured prices already reflected promotions, they reflect the prices paid by consumers; where they did not, the estimates may be conservative, as less healthy items are more likely to be featured through promotional strategies, such as combination deals and volume incentives (Huang *et al*. 2025, Morrison *et al*. 2026). Finally, the data describe menus rather than transactions and contain no purchaser-level information, so the observed price gradients cannot be linked to individual consumers.

### Future research and policy recommendations

Future research should build on this work by incorporating sales data to quantify the effects of supply-side pricing structures and temporary promotions on real-world purchasing behaviour. In particular, linking menu-level price and calorie data to household purchasing panels, such as Worldpanel (formerly Kantar), would allow bundles, set meals, and complete orders to be analysed as consumers actually buy them. Menu data alone cannot do this: a multi-item order has no meaningful per-portion price or calorie value until it is matched to a complete order. Furthermore, as web-based ordering grows and tools for collecting data improve, future studies should also expand to larger datasets to examine different types of pricing incentives as well as children’s menus and items marketed as healthy, regardless of their actual nutritional quality. Comparable per-energy pricing analyses of physical (bricks-and-mortar) takeaway and restaurant outlets would provide a more direct comparison than the retail prices used here. Such per-energy cost analyses have been conducted for physical fast-food outlets elsewhere (Wellard *et al*. 2015), but we are not aware of a comparable UK analysis of the price per unit of energy for meals from physical OOH outlets; UK studies to date have characterized their energy content rather than price (Robinson *et al*. 2018, Muc *et al*. 2019).

With price a major driver of food choice (Darmon and Drewnowski 2015), the identification of price incentives that disadvantage lower-calorie options suggests that structural policy interventions addressing market pricing mechanisms may be warranted.

Evidence from intervention studies in retail and controlled settings suggests that aligning market incentives with public health goals can influence consumer choices, including price reductions (Huangfu *et al*. 2024), targeted subsidies for healthier food (Comini *et al.* 2025), and taxation of less healthy items as is the case for drinks through the Soft Drinks Industry Levy (Rogers *et al*. 2023).

Translating this retail-based evidence to the digital food environment requires adapting mechanisms to platform-specific structures. Because digital platforms embed discounts and bundles directly into the ordering interface, interventions targeting these promotional mechanisms can affect consumer choices. Potential strategies therefore include proportional pricing (Finlay *et al*. 2023) and fiscal or regulatory approaches targeting digital price promotions, with the aim of weakening the financial advantage currently attached to higher-calorie options. Furthermore, comprehensive interventions that combine taxation of unhealthy foods with subsidies for healthier alternatives may be more effective than taxes alone (Barry *et al*. 2023, Pineda *et al*. 2024).

Beyond fiscal measures, corporate accountability frameworks offer another avenue for structural intervention. For instance, under the UK Government’s recently announced ‘healthy food standard’, large food businesses would be required to report on the healthiness of their sales and to meet mandatory targets for improving it (Department of Health and Social Care 2025). Extending this mandatory accountability framework to the digital food environment could encourage platforms to address their pricing structures by using targeted discounts for healthier options to counteract the per-calorie pricing gradient.

## Conclusion

This analysis of menu data from two major OFD platforms in the UK provides evidence of two distinct pricing patterns: a nonlinear relationship, whereby the cost per calorie decreases as calories increase, and a relative price premium for lower-calorie items. Together, these patterns may incentivize purchasing higher-calorie items while posing an economic barrier to selecting lower-calorie options. Addressing these misaligned incentives through structural policy interventions may be warranted to mitigate diet-related health inequalities within the digital food environment.

## Supplementary Material

daag126_Supplementary_Data

## Data Availability

Due to its nature, we cannot share the data used in this study. However, the automated tools used for data collection are publicly available and referenced.
