# Supplementary material for: Is there a healthy-food premium? A cross-sectional analysis of the UK online food delivery sector
Source: Health Promot Int. 2026 Aug 17;41(4):daag126. doi: 10.1093/heapro/daag126 (PMC13479319; doi:10.1093/heapro/daag126)
Supplement: Supplementary file 1 [file daag126_supplementary_data.docx]

**Supplementary File**

**Appendix A: List of keywords used for menu item re-categorisation**

| **Category** | **Keywords** |
| --- | --- |
| Burger | burger, cheeseburger, bacon burger, smash burger, quarter pounder, big mac, mcbacon, mcchicken, mcdouble, whopper, fillet-o-fish, veggie burger, vegan burger, halloumi burger |
| Pizza | pizza, margherita, pepperoni, hawaiian, veggie supreme, deep pan, thin crust, calzone, sourdough pizza, four cheese, sicilian, napoli |
| Sandwich | sandwich, wrap, sub, baguette, panini, toastie, blt, club sandwich, ciabatta, hoagie, melt, slider, philly cheesesteak, croque, sarnie, schiacciata |
| Taco | taco, tacos, al pastor, barbacoa, carnitas, soft taco, hard taco |
| Curry | curry, korma, masala, tikka masala, jalfrezi, balti, rogan josh, bhuna, vindaloo, madras, dhansak, pathia, pasanda, ceylon, katsu curry, thai green curry, thai red curry, massaman |
| Kebab | kebab, doner, donner, shish, kofta, kofte, gyro, shawarma, mixed kebab, chicken kebab, lamb kebab |
| Fried Chicken | fried chicken, chicken wing, hot wing, spicy wing, boneless wing, boneless, chicken strip, chicken tender, chicken select, nugget, popcorn chicken, karaage |
| Steak & Grill | steak, grill, sirloin, ribeye, t-bone, filet mignon, rump steak, gammon, grilled chicken, jerk chicken, harissa chicken, charred chicken, lamb chop, pork chop, bangers & mash |
| Stir Fry | stir-fry, kung pao, black bean sauce, szechuan, teriyaki, cashew nut, oyster sauce, crispy shredded beef |
| Noodle & Pasta | noodle, noodles, chow mein, pad thai, ramen, pho, udon, soba, laksa, singapore noodles, yaki soba, pasta, penne, fusilli, rigatoni, fettuccine, spaghetti, bolognese, carbonara, linguine, tagliatelle, mac & cheese |
| Rice Dish | rice, fried rice, special fried rice, egg fried rice, biryani, pilau, paella, risotto, jollof, kedgeree |
| Seafood | seafood, fish, prawn, shrimp, salmon, cod, haddock, tuna, sea bass, scampi, calamari, mussel, crab, lobster, fish and chips |
| Pie & Pastry | pie, pasty, steak and ale, chicken and mushroom, shepherd's pie, cottage pie, cornish pasty, sausage roll, beef wellington |
| Salad | salad, caesar, nicoise, cobb, greek salad, caprese, waldorf, poke bowl, build your own bowl, buddha bowl |
| Soup | soup, chowder, bisque, consomme, goulash, miso, tom yum, lentil soup, minestrone |
| Sides | fries, chips, potato wedges, tater tots, hash brown, mashed potato, garlic bread, pitta, naan, roti, dough ball, breadstick, onion rings, mozzarella stick, jalapeno popper, onion bhaji, samosa, spring roll, prawn toast, pakora, aloo tikki, coleslaw, side salad, corn on the cob, hummus, tzatziki, edamame, cucumber |
| Toppings & Extras | topping, extra, add-on, additional, supplement, cheese topping, bacon topping, mushrooms topping, pepperoni topping, sauce extra, dip extra, condiment, garnish, sprinkle, drizzle |
| Bundle | bundle, combo, meal deal, set meal, for two, for 2, spice bag |
| Sharing | sharing, share, shared, family size, family bucket, large portion, big portion, extra large, xl size, group meal, party size, feast, banquet, platter, tray, board, spread |
| Breakfast | breakfast, brunch, full english, fry up, eggs benedict, royale, florentine, omelette, scrambled eggs, pancakes, french toast, porridge, oatmeal, bacon bap, croissant |
| Kids | kids, children's, junior, little one, happy meal, mighty meal, child, mini, small portion, kids' menu, children's menu, junior menu |
| Dessert | dessert, sweet, cake, cheesecake, brownie, cookie, ice cream, sundae, waffle, waffles, crepe, pudding, tiramisu, mousse, doughnut, knafeh, kunafa, kanafeh, chocolate bar |
| Drink | drink, coke, coca-cola, pepsi, diet coke, diet pepsi, zero, fanta, sprite, 7up, tango, juice, water, sparkling, still, coffee, tea, latte, cappuccino, beer, wine, white wine, red wine |

The subcategories identified above were initially collapsed into ten overarching categories. Specifically, 13 subcategories (e.g., burgers, pizzas, curries) were aggregated into Main Dishes, and breakfasts were grouped under Bundles & Set Meals. The remaining subcategories mapped directly to their correspondingly named main categories.

**Appendix B: Analytical strategy: The relationship between price and calories**

Model Equation (Fixed-effects model with quadratic term):

$$Y_{ijm}=\beta_{0}+\beta_{1}\cdot\text{kca}\text{l}_{\text{i}}+\beta_{2}\cdot\text{kca}\text{l}_{\text{i}}^{\text{2}}+\delta_{m}+\alpha_{j}+\epsilon_{ijm}$$

- *Yᵢⱼₘ*: the absolute price (in pence) of item $i$ in Item category $m$ at restaurant chain $j$.
- kcal*ᵢ*, kcal*ᵢ*²: the calorie content of item $i$ (scaled to 100-kcal units).
- δ*ₘ*: Item category fixed effects.
- α*ⱼ*: restaurant-chain fixed effects.
- ε*ᵢⱼₘ*: error term.

To investigate heterogeneity by item category, the model was extended to include interaction terms between calories and item categories:

Model Equation:

$$Y_{ijm}=\beta_{0}+\sum_{m} \left( \beta_{1m}\text{kcal}_{i}\times I_{m} \right)+\sum_{m} \left( {\beta_{2m}\text{kcal}}_{i}^{2}\times I_{m} \right)+\delta_{m}+\alpha_{j}+\varepsilon_{ijm}$$

- *Iₘ*: Indicator variable for item category *m*.
- *β₁ₘ, β₂ₘ*: Category-specific coefficients for the linear and quadratic calorie terms, respectively.

Heterogeneity across categories was evaluated via a joint F-test of the interaction terms.

**Appendix C: Analytical strategy: Recommended calorie indicator and price differences**

Model Equation (Fixed-effects model):

$$Y_{ijm}=\beta_{0}+\beta_{1}\text{Indicator}_{i}+\delta_{m}+\alpha_{j}+\varepsilon_{ijm}$$

- *Yᵢⱼₘ*: the price per 100 kcal (in pence) of item $i$ in Item category $m$ at restaurant chain $j$.
- *Indicatorᵢ*: binary variable taking the value 1 if item *i* meets the recommended calorie threshold (<600 kcal), and 0 otherwise.

To assess whether price differences varied by category, the following interaction model was estimated:

Model Equation:

$$Y_{ijm}=\beta_{0}+\sum_{m} \left( \beta_{1m}\text{Indicator}_{i}\times I_{m} \right)+\delta_{m}+\alpha_{j}+\varepsilon_{ijm}$$

- *β₁ₘ*: Category-specific price difference associated with the recommended calorie indicator.

Heterogeneity was evaluated via a joint F-test of the interaction terms.

**Appendix D: Main effects model for recommended calorie indicator**

| **Variable** | **Coefficient** | **Robust SE** | **95% CI** | **P-value** |
| --- | --- | --- | --- | --- |
| **Items meeting calorie recommendations  (< 600 kcal)** | **70.34** | **4.30** | **[61.87, 78.81]** | **< 0.001** |
| **Item Categories  (Ref: Desserts)** |  |  |  |  |
| Main Dishes | 42.02 | 5.22 | [31.74, 52.31] | < 0.001 |
| Sides & Snacks | 1.20 | 6.93 | [-12.48, 14.87] | 0.863 |
| Salads & Bowls | 84.51 | 10.71 | [63.39, 105.63] | < 0.001 |
| Soups | 90.44 | 17.71 | [55.52, 125.37] | < 0.001 |
| Other | 28.33 | 6.71 | [15.09, 41.57] | < 0.001 |
| Constant | 88.42 | 5.20 | [78.16, 98.67] | < 0.001 |

Note: Dependent variable: price per 100 kcal (pence). The model includes restaurant-chain and item category fixed effects with standard errors clustered by chain. Item category coefficients represent differences from the reference category (Desserts). "Items meeting calorie recommendations" is defined as items containing <600 kcal. N = 7,392; R² = 0.429.

**Appendix E: Heterogeneity in the price-calorie relationship by item category**

| **Variable** | **Coefficient** | **Robust SE** | **95% CI** | **P-value** |
| --- | --- | --- | --- | --- |
| Calories  (linear term for Ref) | **45.59** | **8.20** | **[29.42, 61.76]** | **< 0.001** |
|  |  |  |  |  |
| Item Categories  (Ref: Desserts) |  |  |  |  |
| Main Dishes | -32.52 | 35.24 | [-102.02, 36.98] | 0.357 |
| Sides & Snacks | -124.13 | 44.15 | [-211.19, -37.07] | 0.005 |
| Salads & Bowls | -43.70 | 68.71 | [-179.19, 91.80] | 0.526 |
| Soups | -3.94 | 89.05 | [-179.54, 171.67] | 0.965 |
| Other | -122.69 | 51.53 | [-224.30, -21.08] | 0.018 |
|  |  |  |  |  |
| Interactions  (Category × Calories) |  |  |  |  |
| Main Dishes × Cal | 76.81 | 11.61 | [53.91, 99.71] | < 0.001 |
| Sides & Snacks × Cal | 34.18 | 12.62 | [9.30, 59.06] | 0.007 |
| Salads & Bowls × Cal | 108.97 | 24.17 | [61.32, 156.62] | < 0.001 |
| Soups × Cal | 93.31 | 47.50 | [-0.35, 186.97] | 0.051 |
| Other × Cal | 56.99 | 16.33 | [24.79, 89.20] | 0.001 |
|  |  |  |  |  |
| Calories²  (squared term for Ref) | -0.32 | 0.45 | [-1.21, 0.58] | 0.486 |
|  |  |  |  |  |
| Interactions  (Category × Calories²) |  |  |  |  |
| Main Dishes × Cal² | -2.69 | 0.65 | [-3.96, -1.42] | < 0.001 |
| Sides & Snacks × Cal² | -0.84 | 0.77 | [-2.36, 0.68] | 0.276 |
| Salads & Bowls × Cal² | -6.39 | 1.72 | [-9.79, -2.99] | < 0.001 |
| Soups × Cal² | -2.41 | 5.92 | [-14.08, 9.26] | 0.684 |
| Other × Cal² | -2.03 | 1.03 | [-4.07, 0.01] | 0.051 |
|  |  |  |  |  |
| Constant | 345.85 | 25.36 | [295.84, 395.87] | < 0.001 |

**Note:** Dependent variable: price in pence. Calories scaled to 100 kcal units. The model includes restaurant-chain and item-category fixed effects with standard errors clustered by chain. Reference category: Desserts. F-test for joint significance of all interaction terms (both linear and squared interactions with categories): F(10, 199) = 9.24, p < 0.001.

**Appendix F: Supplementary results: Heterogeneity of price differences by item category**

| **Variable** | **Coefficient** | **Robust SE** | **95% CI** | | **P-value** |
| --- | --- | --- | --- | --- | --- |
| Items meeting calorie recommendations  (< 600 kcal) | 62.32 | 6.06 | [50.36, 74.28] | | < 0.001 |
|  |  |  |  | |  |
| Item Categories  (Ref: Desserts) |  |  |  |  | |
| Main Dishes | 37.66 | 5.42 | [26.97, 48.36] | | < 0.001 |
| Sides & Snacks | 8.45 | 7.51 | [-6.36, 23.26] | | 0.262 |
| Salads & Bowls | 38.24 | 9.71 | [19.09, 57.39] | | < 0.001 |
| Soups | 38.86 | 16.05 | [7.20, 70.52] | | 0.016 |
| Other | 18.91 | 6.58 | [5.93, 31.89] | | 0.005 |
|  |  |  |  | |  |
| Interactions (< 600 kcal × Category) |  |  |  | |  |
| < 600 kcal × Main Dishes | 5.42 | 7.57 | [-9.52, 20.36] | | 0.475 |
| < 600 kcal × Sides & Snacks | -11.23 | 11.21 | [-33.34, 10.88] | | 0.318 |
| < 600 kcal × Salads & Bowls | 59.52 | 14.98 | [29.98, 89.06] | | < 0.001 |
| < 600 kcal × Soups | 65.09 | 29.39 | [7.13, 123.04] | | 0.028 |
| < 600 kcal × Other | 13.11 | 11.30 | [-9.18, 35.40] | | 0.248 |
|  |  |  |  | |  |
| Constant | 94.03 | 4.77 | [84.64, 103.43] | | < 0.001 |

**Note:** Dependent variable: price per 100 kcal (pence). The model includes restaurant-chain and item-category fixed effects with standard errors clustered by chain. Reference category: Desserts. F-test for joint significance of price differences × category interaction terms: F(5, 199) = 6.35, p < 0.001.

**Appendix G: Sensitivity analysis re-including bundles and sharing items**

| **Sample** | **N** | **Premium for items <600 kcal (pence/100 kcal)**  **[95% CI]** | **Linear term**  **[95% CI]** | **Quadratic term**  **[95% CI]** | **Turning point (kcal)**  **[95% CI]** |
| --- | --- | --- | --- | --- | --- |
| Main analysis (single items) | 7,392 | 70.34  [61.9, 78.8] | 101.14  [87.98, 114.29] | −2.19  [−2.87, −1.50] | 2,309  [1,854, 2,773] |
| Combined (single items + bundles + sharing) | 8,667 | 74.13  [64.7, 83.5] | 93.55 [79.53, 107.57] | −1.47 [−2.15, −0.80] | 3,174 [2,121, 4,227] |
| Bundles and sharing items only | 1,275 | 86.99  [49.5, 124.5] | 80.64  [27.18, 134.11] | −0.56 (n.s.)  [−2.42, 1.31] | — |

Note: Two models were estimated for each sample: the premium column is from a main-effects model of price per 100 kcal (pence) on an indicator for items <600 kcal; the linear term, quadratic term and turning point are from a quadratic model of absolute item price (pence) on calorie content (per 100 kcal) and its square. Both models include menu-category and restaurant-chain fixed effects, with standard errors clustered by chain. 95% confidence intervals are shown in brackets (delta method for the turning points). The price premium for items below 600 kcal persisted in all three samples; for bundles and sharing items only, the quadratic term was not significant and no turning point is defined.

**Appendix H: Contents of the “Other” category**

| **Sub-group** | **Examples (kcal, price)** |
| --- | --- |
| Composite and cross-cuisine dishes | pulled pork nachos (843 kcal, £9.00); aubergine and chickpea tagine (706 kcal, £13.25); chicken quesadilla (633 kcal, £8.25); chorizo antipasti (240 kcal, £3.90); beef empanada (236 kcal, £6.25) |
| Sauces and gravies | béarnaise sauce (245 kcal, £2.50); peppercorn sauce (114 kcal, £2.50); regular gravy (60 kcal, £1.99) |
| Dips and cold sauces | aioli (324 kcal, £1.00); guacamole (216 kcal, £1.99); sour cream (95 kcal, £1.00); salsa (98 kcal, £1.99) |
| Condiment pots, dressings and chutneys | pot of mayonnaise (392 kcal, £1.00); pot of ketchup (58 kcal, £1.00); bleu cheese dressing (69 kcal, £1.25); chilli chutney (197 kcal, £6.50) |
| Olives and pickles | mixed olives (139 kcal, £4.10); nocellara olives (135 kcal, £3.50); carrot and chilli pickle (123 kcal, £2.50) |

Note: The “Other” category is a heterogeneous residual of items that the deterministic keyword rules did not assign to any single standard category — comprising both composite or cross-cuisine dishes and stand-alone accompaniments (sauces, dips, condiments and pickles). It is a residual grouping rather than a claim about any single food type; the examples shown are illustrative and not exhaustive.

**Appendix I: Examples of items exceeding the 2,500 kcal exclusion threshold**

| **Sub-group** | **Examples (kcal, price)** |
| --- | --- |
| Sharing items and multi-person set meals (n = 97) | 50-piece cookie box (9,421 kcal, £25.59); sharing set menu for four (8,203 kcal, £139.99); mixed-grill party platter (7,550 kcal, £35.99); family fried-chicken box (7,420 kcal, £42.00); chicken party bucket (3,360 kcal, £39.99); pizza sharing box (3,705 kcal, £9.20) |
| Very large single products (n = 21) | large loaded pepperoni pizza (3,707 kcal, £28.98); “mega” double-pepperoni pizza (3,105 kcal, £18.49); whole grilled chicken (2,895 kcal, £18.00); large meat-feast pizza (2,791 kcal, £15.99) |

Note: These items were excluded from the analytical sample as extreme-calorie outliers (≥ 2,500 kcal; n = 118). The great majority were sharing items or multi-person set-meal bundles (n = 97); the remainder were very large single products, predominantly family-sized pizzas (n = 21). The examples shown are illustrative and not exhaustive.
